# Effects of Phenolic Pollution on Interspecific Competition between *Microcystis aeruginosa* and *Chlorella pyrenoidosa* and their Photosynthetic Responses

**DOI:** 10.3390/ijerph16203947

**Published:** 2019-10-17

**Authors:** Xiao Tan, Kaiwen Dai, Keshab Parajuli, Xiaoshuai Hang, Zhipeng Duan, Yue Hu

**Affiliations:** 1 Key Laboratory of Integrated Regulation and Resource Development on Shallow Lakes, Ministry of Education, College of Environment, Hohai University, Nanjing 210098, China; algaegroup@163.com (K.D.); duanzhipeng@hhu.edu.cn (Z.D.); 1714060112@hhu.edu.cn (Y.H.); 2 School of Population and Global Health, Faculty of Medicine, Denistry and Health Sciences, The University of Melbourne, VIC 3010 Melbourne, Australia; kparajuli@student.unimelb.edu.au; 3 Nanjing Institute of Environmental Sciences, Ministry of Ecology and Environment, Nanjing 210042, China; hxs@nies.org

**Keywords:** phenol, cyanobacteria, green algae, interspecific competition, Lotka-Volterra model, dominance establishment, photosynthetic activity

## Abstract

The demand for phenolic compounds has been increasing rapidly, which has intensified the production and usage of phenol at a commercial scale. In some polluted water bodies, phenol has become one of the typical aromatic contaminants. Such water bodies are inescapably influenced by nutrients from human activities, and also suffer from nuisance cyanobacterial blooms. While phenolic pollution threatens water safety and ecological balance, algal cells are ubiquitous and sensitive to pollutants. Therefore, effects of phenolic pollution on interspecific competition between a bloom-forming cyanobacterium and other common alga merit quantitative investigation. In this study, the effects of phenol on *Microcystis aeruginosa* (*M. aeruginosa*, a bloom-forming cyanobacterium) and *Chlorella pyrenoidosa* (*C. pyrenoidosa*, a ubiquitous green alga) were analyzed in mono- and co-cultures. The two species were exposed to a series of phenol treatments (0, 2, 20, and 200 μg mL^−1^). Population dynamics were measured by a flow cytometer and analyzed by the Lotka-Volterra model. The results showed that *M. aeruginosa* was more sensitive to phenol (EC_50_ = 80.8 ± 0.16 μg mL^−1^) compared to *C. pyrenoidosa* (EC_50_ = 631.4 ± 0.41 μg mL^−1^) in mono-cultures. *M. aeruginosa* won in the co-cultures when phenol was below or equal to 20 μg mL^−1^, while *C. pyrenoidosa* became the dominant species in the 200 μg mL^−1^ treatment. Photosynthetic activity was measured by a fluometer. Results showed phenol significantly impacted the photosynthetic activity of *M. aeruginosa* by inhibiting the acceptor side of its photosystem II (PSII), while such inhibition in *C. pyrenoidosa* was only observed in the highest phenol treatment (200 μg mL^−1^). This study provides a better understanding for predicting the succession of algal community structure in water bodies susceptible to phenolic contamination. Moreover, it reveals the mechanism on photosynthetic responses of these two species under phenolic stress.

**Highlights:***M. aeruginosa* was dominant in co-cultures with *C. pyrenoidosa* at a lower concentration of phenol (below or equal to 20 μg mL^−1^). A higher concentration of phenolic pollution (up to 200 μg mL^−1^) facilitated the dominance of *C. pyrenoidosa* in co-cultures with *M. aeruginosa*. Phenol inhibits the acceptor side of electron transport chain and reduces the number of active reaction centers of photosystem II in *M. aeruginosa* cells.

## 1. Introduction

Phenol is a common aromatic organic compound in surface waters [1,2,3], and mainly originates with wastewaters from various industries, including the coal mining, phenol production, and textile industries [4,5]. In China, phenol is generally at 0.2 to 12 μg L^−1^ in unpolluted lakes [6,7]. However, its concentration can be much higher in wastewater from petroleum refineries (13–88 μg mL^−1^) and in coke wastewater (180 μg mL^−1^) from steel facilities [3]. In 2005, in Tonglu County (China), the concentration of phenol reached 243 μg mL^−1^ in the polluted water area [8]. More recently, with the increasing production and usage of phenol, large numbers of lakes and rivers suffer from phenolic pollution.

The presence of phenol in water bodies generally poses severe risks to human health (corrosive and carcinogenic toxicity) and water safety. Moreover, phenolic pollution can threaten the balance of aquatic ecosystems. Algal cells are ubiquitous, and are sensitive to aromatic pollutants. Therefore, greater insights into their damaging effects on phytoplankton are crucial, given their role on primary productivity in ecosystems. Several studies have reported that the structure of algal cells can be affected by the existence of phenol; for instance, the membranes of algal cells may be damaged by hydrophobic interaction with lipid bilayer structures [9,10]. Additionally, photosynthetic systems of algal cells might be damaged by phenol, as recent studies have shown that phenol changes their PSII structures and functions [10,11]. However, most studies have focused on the effects of phenol on the growth and physiology of single algal species in mono-cultures [11,12,13], while there is a lack of information on its influence on interspecific competition and dominance establishment. Such knowledge is of critical significance in relation to predicting and protecting the stability of aquatic ecosystems.

In recent years, some studies have shown that organic pollutants (such as alkylbenzene sulfonate and pentachlorophenol) have the potential to overturn algal community structure [14,15]. For example, *M. aeruginosa* competed over *Scenedesmus obliquus* in co-cultures without alkylbenzene sulfonate (LAS), while the result was opposite when LAS (20 μg mL^−1^) was added in co-cultures [15]. Similarly, the effects of pentachlorophenol (PCP) on *M. aeruginosa* and *Chlorella vulgaris* were studied in co-cultures [16]. Results showed that the effect of PCP on *M. aeruginosa* was concentration-specific, where low levels of PCP promoted the growth of *M. aeruginosa*, while high concentrations of PCP significantly inhibited its growth. However, no toxic effect of PCP on *C*. *vulgaris* was observed. This suggests that exogenous pollutants can affect the interspecific competition between *M. aeruginosa* and *S. obliquus*, thereby influencing the community structure. Moreover, it has been reported that polycyclic aromatic hydrocarbons (PAHs) could also affect the algal interspecific competition [14,17]. Despite the potential influences of organic pollutants on the interspecific relationships, the mechanism involved in the competitive process still remain open for further investigation, which merits quantitative analysis to better understand such phenomena under polluted conditions.

Cyanobacterial blooms threaten drinking water supplies, fisheries, and recreational activities. Competition between cyanobacteria and green algae affects phytoplankton succession and the formation of blooms [18]. In China, *Microcystis* is a very common bloom-forming cyanobacterium. Some organic compounds could influence the growth of *Microcystis* [15,19], while the detailed information about the competition between *Microcystis* and other algae under the stress of phenol is still unclear. Therefore, in this study, two common species of phytoplankton (*M. aeruginosa* and *C. pyrenoidosa*) were chosen to investigate the effects of phenol in mono- and co-cultures. Their growth and photosynthetic parameters were measured to analyze their competitive relationships and photosynthetic responses to various concentrations of phenol. We aimed to provide some useful information for predicting the succession of algal community structure in water bodies susceptible to phenolic contamination.

## 2. Materials and Methods

### 2.1. Preparation of Chemical Reagents

Phenol was purchased from Aladdin Industrial Corporation of Shanghai, with a purity > 99.0%, which was pre-dissolved in dimethyl sulfoxide (DMSO). The concentration of DMSO was lower than 0.5 mL L^−1^ to ensure DMSO was nontoxic to algal cells [20]. The phenol-DMSO solution was stored in an airtight bottle and was protected from light.

### 2.2. Strains and Culture Conditions

*M. aeruginosa* (PCC-7806) and *C. pyrenoidosa* (FACHB-11) were purchased from the Institute of Hydrobiology, Chinese Academy of Science. The two strains were cultured in sterilized BG11 media [21] at 25 ± 0.5 °C, with a light: dark cycle of 12 h: 12 h at 65 μmol photons m^−2^ s^−1^ (FSL, China). Algal cells in the mid-exponential growth phase were used for the experiments.

### 2.3. Experimental Design

To evaluate the effects of phenol on growth, photosynthesis, and interspecific competition between *M. aeruginosa* and *C. pyrenoidosa*, their mono-cultures and co-cultures were designed. Experiments were carried out in 250 mL Erlenmeyer flasks containing 100 mL algal suspension. Phenol was set at four treatment groups (0, 2, 20, and 200 μg mL^−1^), to reflect its concentrations in polluted water bodies in China mentioned above. In this study, the initial biomass ratio of *M. aeruginosa* to *C. pyrenoidosa* was set at 1:1, and the initial cell concentration was adjusted based on the volume of individual cells [22]. Therefore, the cell concentrations of *M. aeruginosa* and *C. pyrenoidosa* were set at 1.0 × 10^5^ and 2.0 × 10^5^ cells mL^−1^ at the beginning, respectively. Experiments were carried out in triplicate.

### 2.4. Cell Counting

Cells of the two species are similar in size and morphology, which are difficult to be distinguished and enumerated accurately by an optical microscope. Therefore, flow cytometry (Accuri C6 plus, BD) was employed to analyze cell concentrations every two days. Replicated samples (2 mL) from the different treatment groups were analyzed. For the quantification of cell concentration, an aliquot of a calibrated solution of fluorescent beads (1 μm diameter, BD) was added in each sample as an internal standard. Two species were differentiated by auto-fluorescent signals of chlorophyll and phycocyanin.

### 2.5. Measurement of In Vivo Chlorophyll-a Fluorescence

To monitor photosynthetic activity responses to phenol, photosynthetic efficiencies of monocultured *M. aeruginosa* and *C. pyrenoidosa* were determined by in vivo chlorophyll-a (Chl *a*) fluorescence. Firstly, 2 mL of algal cells were kept in the dark for 15 min. Then a series of chlorophyll-a fluorescence parameters and the induction curves were recorded by a FluorPen fluorometer (AquaPen C100, Photon Systems Instruments, Czech) at room temperature [23,24,25]. This fluorometer was equipped with two types of excitation wavelength at 455 nm and 620 nm. The OJIP test (Fluorescence response curves detection) was conducted to analyze the changes in these fluorescent parameters, which can provide adequate information about the structure, conformation, and the function of photosynthetic apparatus. From the OJIP transient, the measured parameters (*F_0_*, *F_m_*, *F_300μs_*, *F_J_*, *F_I_* and so on) were used to calculate new parameters, which are displayed in Table 1 [24,25].

### 2.6. Measurement of Dissolved Phenol Concentration

The concentration of dissolved phenol was determined every two days after filtration (using GF/F membrane, Whatman) based on the standard method [26]. Phenol concentrations were measured via a spectrophotometer (Shimadzu UV-2450, Japan) after chloroform extraction. The absorbance of the colored complex of phenol with 4- amino antipyrine was detected at 460 nm [26]. Moreover, to detect the abiotic degradation of phenol during the experiment, a blank control (BG11 medium with phenol and without algal cells) was designed to measure the concentration of phenol every two days.

### 2.7. Statistical Analyses

Statistical analyses were performed using SPSS 19.0 (IBM, Armonk, NY, USA). One-way analysis of variance (ANOVA) was applied to determine the significance of differences among the different treatments and least significant difference (LSD) multiple comparison was performed (*p* < 0.05 was considered as the level of significance). Data were expressed as mean ± standard deviation (SD). Logistic growth model was used to fit the data and describe the population dynamics of *M. aeruginosa* and *C. pyrenoidosa*.

Based on the growth curves, half-maximal effective concentration (EC_50_) was estimated at 96 h in terms of interpolated concentration that would inhibit growth by 50% over a specific period (96 h). EC_50_ was estimated using a dose-response model for probit analysis on the fourth day [27].

Growth curves were fitted according to the following logistic function:

(1) $$N_{\left(t\right)}=K/(1+e^{a-rt})$$

where *N*_(*t*)_ is the cell concentration (10^5^ cells mL^−1^) at t; *K* is the carrying capacity (10^5^ cells mL^−1^) of the population, defined as maximum sustainable population biomass in a given space; *a* is the coefficient indicating the intercept of growth curves; *r* is the intrinsic growth rate, and *t* is the duration of culture [14,17].

The occurrence of inflection on growth curves reflects the initiation of inhibition, which was calculated using the following formula:

(2) $$T_{p}=a/r$$

where *T_p_* is the initiation of inhibition, *a* is coefficient indicating the intercept of the growth curves, and *r* is the intrinsic growth rate.

The Lotka-Volterra competitive model [28] was used to calculate the population dynamics of the two species under the stress of organic compounds in co-cultures [14,15], which was calculated using the following formula:

(3) $$\frac{Nmn-Nmn-1}{tn-tn-1}=\frac{rmnNmn-1\left(Kmn-Nmn-1-\alpha Ncn-1\right)}{Kmn}$$

(4) $$\frac{Ncn-Ncn-1}{tn-tn-1}=\frac{rcnNcn-1\left(Kcn-Ncn-1-\beta Nmn-1\right)}{Kcn}$$

where *N_mn_* (*N_mn_*_−*1*_) and *N_cn_* (*N_cn_*_−*1*_) represent the cell concentrations of *M. aeruginosa* and *C. pyrenoidosa*, respectively, when they were co-cultured at day *t_n_* (*t_n_*_−*1*_); *r_mn_* and *r_cn_* are the intrinsic growth rates of *M. aeruginosa* and *C. pyrenoidosa*, respectively, which are calculated according to the mono-cultures; *K_mn_* and *K_cn_* are the carrying capacity of each unit of cell concentrations of *M. aeruginosa* and *C. pyrenoidosa* respectively in mono-cultures; *α* and *β* are the competition coefficients in co-cultures; *α* indicates the inhibition of *C. pyrenoidosa* on *M. aeruginosa*; and *β* represents the inhibition of *M. aeruginosa* on *C. pyrenoidosa*.

## 3. Results and Discussion

### 3.1. Effects of Phenol on Algal Growth in Mono- and Co-Cultures

In this study, data were calculated and fitted by the equation to estimate the EC_50_ on the fourth day, because aromatic pollutants decreased the population growth and biomass of microalgae after three days [27]. Therefore, the endpoint biomass measurement for dose-response analysis after 96 h exposure was more convincing. Growth rates varied with species and culture types after 96 h of exposure to phenol, and the EC_50_ of *M. aeruginosa* was 80.8 ± 0.16 μg mL^−1^ in mono-cultures, which dropped to 54.7 ± 0.25 μg mL^−1^ in co-cultures (Table 2). This reflected that interspecific competition increased the toxicant sensitivity of *M. aeruginosa* to phenol [29,30]. However, *C. pyrenoidosa* showed high resistance to phenol, as its EC_50_ was as high as 565.9 ± 0.41 μg mL^−1^ in mono-cultures. A previous work studied four nitrophenolics (*o*-nitrophenol, *p*-nitrophenol, *m*-nitrophenol, 2, 4-dinitrosophenol) toward the growth of *C*. *vulgaris* and two cyanobacteria (*Nostoc muscorum* and *Nostoc linckia*), and the EC_50_ of *C*. *vulgaris* was in the range of 55 to 128 μg mL^−1^, while the EC_50_ values of two cyanobacteria were 32 to 82 μg mL^−1^ [31]. These data were similar to our results, but the nitrophenolic compound is more toxic than phenol to *Chlorella* sp..

As for quantitative analyses, *M. aeruginosa* unicells were much more accessible for enumeration than colonies. Population dynamics and interspecific competition between the two species can be analyzed accurately by flow cytometry and ecological models. It was the reason unicellular *M. aeruginosa* was chosen for this study.

**Figure 1:**
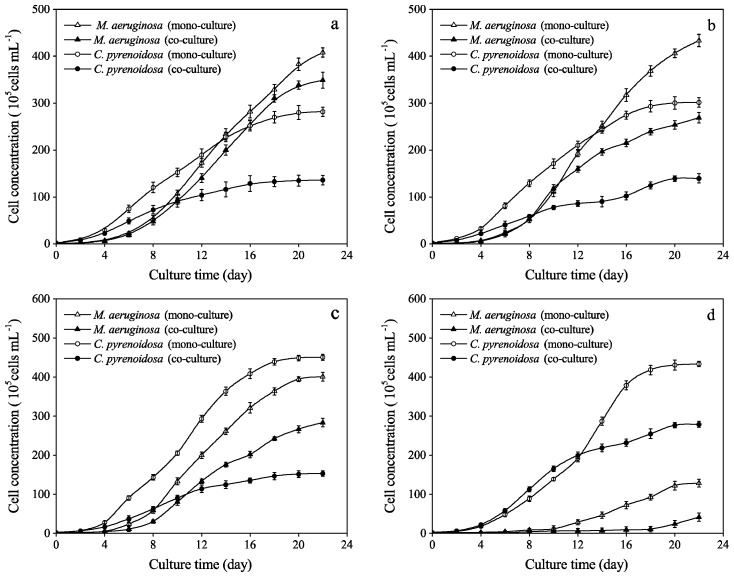
Growth curves of *M. aeruginosa* and *C.pyrenoidosa* in mono-cultures and co-cultures under different treatments of phenol: (**a**) 0 μg mL^−1^, (**b**) 2 μg mL^−1^, (**c**) 20 μg mL^−1^, and (**d**) 200 μg mL^−1^.

Growth curves of *M. aeruginosa* and *C. pyrenoidosa* in mono- and co-cultures are displayed in Figure 1. In mono-cultures, the growth of *M. aeruginosa* was markedly inhibited by high concentration (200 μg mL^−1^) of phenol in the beginning (Figure 1d), while the cell concentration of *C. pyrenoidosa* increased by 61% and 54% in 20 and 200 μg mL^−1^ treatment groups, respectively (Figure 1c,d). Growth hormesis was observed in some algae after exposure to chemicals, based on the findings of previous studies that conducted the dose-response experiments [12,32]. Hormesis is a term for the stimulatory effects caused by a low concentration of the toxic agent. Hormesis phenomenon in *M. aeruginosa* was also observed in this study at low concentrations of phenol, but it exhibited a dose-response effect when phenolic concentration increased, showing a significant inhibition in growth rate. However, *C. pyrenoidosa* grew well despite the increase in phenolic concentration. The ecotoxicity of phenol occurs from the damage to cell membranes via hydrophobic interaction with lipid bilayer structures [33], and phenol could also penetrate cells and damage inner systems, such as endoplasmic reticulum, nuclei, and their components [10]. Moreover, the reactivity of phenol with biomolecules is related to the ease with which it donates free electrons to oxidized substrates and the oxidative stress caused by free radicals and reactive oxygen species (ROS), such as superoxide radicals or hydrogen peroxide [10].

In co-cultures, the growth of *M. aeruginosa* was inhibited even at low concentration (20 μg mL^−1^) of phenol, and its EC_50_ is shown in Table 2. After 22 days, *M. aeruginosa* established dominance in 0, 2, and 20 μg mL^−1^ treatment groups. In contrast, cell concentrations of *C. pyrenoidosa* were significantly higher than those in the control in all but the 2 μg mL^−1^ treatment group. Moreover, its cell concentration in 200 μg mL^−1^ group was about twice that in the co-cultures without phenol. Thus, *C. pyrenoidosa* showed high resistance and strong competitiveness under phenolic stress (Figure 1).

### 3.2. Growth Parameters and Inflection Points

Growth parameters of *M. aeruginosa* and *C. pyrenoidosa* in mono-cultures and co-cultures are presented in Table 3. Given that the coefficient of determination (R^2^) exceeded 0.9 for each treatment for both culture types, it implies that the logistic equation (Equation (1)) nicely fitted the growth curves in Figure 1. The carrying capacity (*K*), intrinsic growth rate (*r*), and initiation of inhibition (*T_p_*) of *C. pyrenoidosa* in co-cultures were lower than those in mono-cultures. For *M. aeruginosa*, the carrying capacity (*K*) and the intrinsic growth rate (*r*) in mono-cultures were higher than those in co-cultures of all groups (Table 3). However, the *T_p_* for *M. aeruginosa* in co-cultures appeared sooner than that in mono-cultures (Table 3).

In co-cultures, the competitive inhibition parameters *α* (*C. pyrenoidosa* against *M. aeruginosa*) and *β* (*M. aeruginosa* against *C. pyrenoidosa*) were calculated based on the data in Table 3, according to Equations (3) and (4). Averages of the competitive inhibition parameters after the occurrence of inflection points are shown in Table 4. The value of *β* was 1.75 ± 0.07 (while *α* was -1.26 ± 0.12) in the co-cultures with no phenol. Interestingly, *β* slightly dropped when phenol was added. Furthermore, when phenol reached 20 μg mL^−1^, *β* was below zero. This indicated that the interspecific competition between *M. aeruginosa* and *C. pyrenoidosa* had been altered by phenol. In co-cultures, *M. aeruginosa* established dominance in 0, 2, and 20 μg mL^−1^ treatment groups, but *C. pyrenoidosa* became the dominant species in the 200 μg mL^−1^ group. In the low-concentration groups (0, 2, and 20 μg mL^−1^), phenol concentration was lower than EC_50_ of *M. aeruginosa* (80.8 ± 0.16 μg mL^−1^) and far less than EC_50_ of *C. pyrenoidosa* (565.9 ± 0.41 μg mL^−1^). Under these conditions, *M. aeruginosa* could maintain a higher growth rate and longer exponential phase compared with *C. pyrenoidosa* (Figure 1 and Table 3). While, in the highest group (200 μg mL^−1^), phenol inhibited the growth of *M. aeruginosa* markedly, but *C. pyrenoidosa* vigorously resisted phenolic stress. Phenol could be metabolized as an organic carbon source for *C. pyrenoidosa* [3]. Thus, a high concentration of phenol overturned their interspecific competition.

### 3.3. Photosynthetic Activities of Two Algae in Mono-Cultures

**Figure 2:**
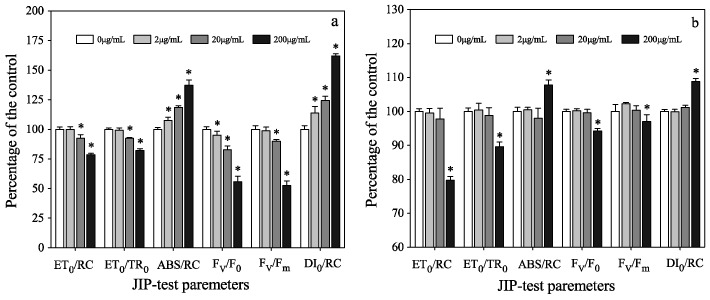
Changes in JIP-test parameters (compared to the control of the same species, and displayed as the percentage of the control) at different concentrations of phenol: (**a**) *M. aeruginosa* and (**b**) *C. pyrenoidosa*. The significant difference compared to the control is indicated as * (*p* < 0.05).

To further investigate the different responses of two algae to phenol, photosynthetic activities were analyzed in mono-cultures. Algal Chl *a* fluorescence is one of the sensitive, non-invasive, and efficient methods to detect cellular responses to pollutants. Analyses on Chl *a* fluorescence induction curves contributed to the evaluation on photosynthetic electron transport chain, which provides valuable information between the inflow and outflow of energy flux in PSII [34,35,36]. In this study, Chl *a* fluorescence was measured in mono-cultures on the fourth day. JIP-test parameters of *M. aeruginosa* (Figure 2a) showed considerable changes compared to the parameters of *C. pyrenoidosa* (Figure 2b).

*F_v_*/*F_m_* is a useful parameter to indicate the maximal photochemical efficiency of PSII in algal cells after dark adaptation, which is related to the probability that an absorbed photon would be trapped by the reaction center (RC), resulting in the reduction of primary plastoquinone (Q_A_) pool [23]. Values of *F_v_*/*F_m_* in *M. aeruginosa* cells were significantly reduced (*p* < 0.05) in 20 and 200 μg mL^−1^ treatment groups, with the 200 μg mL^−1^ group showing a decreasing of more than 50%. However, for *C. pyrenoidosa*, *F_v_*/*F_m_* value decreased slightly in the 200 μg mL^−1^ group only (Figure 2).

Under normal conditions, reactive oxygen species (ROS) are produced at a lower rate. However, some pollutants lead to a dramatic increase in ROS production, which reduces the number of active reaction centers or causes the light-harvesting complexes (LHCs) to detach from the core of PSII [24,37]. For *M. aeruginosa*, *ABS*/RC, a measurement of the average absorption per active RC or the average amount of absorbing chlorophylls per active RC [23], significantly increased at 200 μg mL^−1^ phenol. It has been suggested that the inactivation of RC can account for the increase of *ABS*/RC [38]. Moreover, we found that the sharpest decrease of *F_v_*/*F_0_* (44%) resulted from the reduction in the ratio of active and inactive reaction centers of PSII (Figure 2a). This might have occurred due to the transformation, which turned some of the active RC to ‘silent RC’ (RC ^si^). The RC ^si^ have two characteristics: (a) these centers can neither reduce Q_A_ nor back transfer their excitation energy to the antenna. Hence the corresponding PSII units do not contribute to the variable fluorescence, and their fluorescence yield constantly remains at low levels and is equal to those of units with open RC; and (b) they are re-activated as soon as the stress that provoked the conformational modification ceases [34]. The percentage of treated algae’s RC^si^ compared to the control group was calculated using the following equation [23].

(5) $${RC}^{si}=[1-\frac{{\left(ABS/RC\right)}^{c}}{\left(ABS/RC\right)}]\times 100\%=\{1-\frac{{\left(M_{0}/V_{j}\right)}^{c}}{\left(M_{0}/V_{j}\right)}\times \frac{\left[1-(F_{0}/F_{m})\right]}{{\left[1-(F_{0}/F_{m})\right]}^{c}}\}\times 100\%$$

where RC is the reaction center (RC in the control is signed with a superscript ‘*c*’, inactive or silent RC is shown with a superscript ‘*si*’). Other terms in the equation are listed in Table 1.

**Figure 3:**
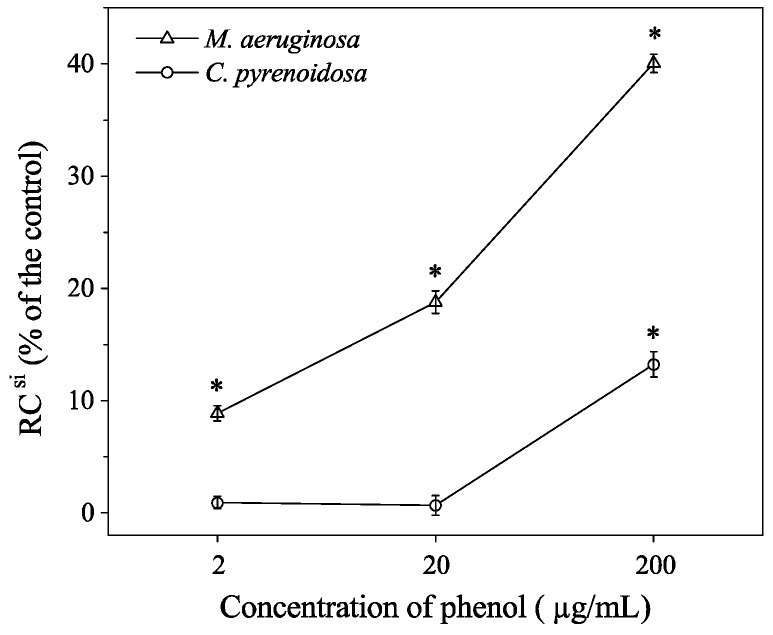
Percentage of the silent reaction center (RC ^si^) at different concentrations of phenol in mono-cultures Significant difference compared with the control is indicated as * (*p* < 0.05).

For *M. aeruginosa*, the percentage of RC ^si^ increased with the concentration of phenol, and there were significant differences between the treatments and the control (*p* < 0.05; Figure 3). Therefore, the reduction of active RC contributed to the decrease of *F_v_*/*F_0_* ratio. Meanwhile, the increased RC^si^ could explain the significant augmentation of *ABS*/RC and *DI_0_*/RC, as it increased by 37% and 62% in the 200 μg mL^−1^ group, respectively (Figure 2a). However, the percentage of RC^si^ in *C. pyrenoidosa* cells only increased by 13.2% in 200 μg mL^−1^ group (Figure 3). Similarly, the electron transport from Q_A_ to Q_B_ flux per RC (*ET_0_*/RC) and the electron transport probability (*ET_0_*/TR_0_) decreased under the stress of phenol [38]. As to *M. aeruginosa*, the *ET_0_*/RC and *ET_0_*/TR_0_ decreased significantly in 20 and 200 μg mL^−1^ groups (Figure 2a). For *C. pyrenoidosa*, there was a significant difference (*p* < 0.05) between the 200 μg mL^−1^ treatment and the control group, as for the 200 μg mL^−1^ group, *ET_0_*/RC and *ET_0_*/*TR_0_* decreased by 20% and 10%, respectively (Figure 2b).

To determine the inhibiting site in the electron transport chain, the fraction of oxygen-evolving complex (OEC) of the treated samples was calculated, and results showed that fraction of OEC remained unchanged in all treated groups. This suggests that electron transport on the donor side of PSII was not affected by phenol. In addition, the decrease of the parameter F_v_/F_0_ also indicates an alteration in the acceptor side of the PSII complex [39]. Therefore, the acceptor side of PSII in electron transport action was inhibited by phenol, which was similar to the detrimental effects of polycyclic aromatic hydrocarbon on wheat [24]. Furthermore, the decrease of *ET_0_*/RC indicated an inhibition on Q_A_^−^, which was similar to artemisinine (act as a kind of algicide to control *Microcystis*), an inhibitor of the electron flow beyond Q_A_^−^ [40,41]. The effects of phenol on PSII were comparable to those of aromatic herbicides (ioxynil). Ioxynil can interact with different amino acid residues on the D1 protein of PSII in cyanobacterium (*Synechocystis salina*), and inhibit the electron transport from Q_A_^−^ to Q_B_ [39].

Moreover, cyanobacterial cells exhibit an internal thylakoid system organized as a series of roughly parallel double-membrane layers distributed within the cytoplasm, whereas green algae have chloroplasts, which contain an inner membrane system formed by bands of stacked thylakoids (grana) and thylakoids running singly in the stroma [39]. It has also been confirmed that there are differences in the polypeptides composition of OEC and peripheral light-harvesting antenna of PSII between cyanobacteria and green algae [42]. These differences can influence photosynthetic activity and toxicant sensitivity to phenolic contamination.

During the experiment, the abiotic degradation or volatilization of phenol was very slow in the blank control during the experiment (Table S1). However, in treatment groups, the measured values were significantly lower than designed values (*p* < 0.01), which was due to the cellular adsorption or digestion by physiological metabolism [3]. Especially in *C. pyrenoidosa* mono-cultures and co-cultures, the dissolved phenol concentrations were markedly lower than those in *M. aeruginosa* mono-cultures (*p* < 0.05). A previous study found that phenol could be metabolized as an organic carbon source for *C. pyrenoidosa* [3]. Further studies will focus on the distribution and digestion of phenol in *C. pyrenoidosa* cells.

## 4. Conclusions

In this study, *M. aeruginosa* established dominance in co-cultures with *C. pyrenoidosa* when phenol was below or equal to 20 μg mL^−1^. However, *C. pyrenoidosa* was the dominant species at 200 μg mL^−1^ of phenol. This means that phenolic pollution could overturn the competition between *M. aeruginosa* and *C. pyrenoidosa*. *M. aeruginosa* was more sensitive to phenol because its photosynthetic activity was inhibited in the acceptor side of electron transport chain and the number of active reaction centers reduced significantly.

## Figures and Tables

**Table 1:** Formulae or terms of the OJIP transient [24,25].

Formulae or terms	Description
*F_0_*	Minimum fluorescence
*F_m_*	Maximum fluorescence
*V_J_* = (*F_2ms_* − *F_0_*)/(*F_m_* − *F_0_*)	Relative variable fluorescence at 2 ms
*M_0_* = 4 (*F_300μs_* − *F_0_*)/(*F_m_* − *F_0_*)	Approximated initial slope (in ms ^−1^) of the fluorescence transient *V* = *f* (*t*); It is a measure of the rate of the primary photochemistry
*V_K_* = (*F_300μs_* − *F_0_*)/(*F_m_* − *F_0_*)	Relative variable fluorescence at 300 μs
*F_v_*/*F_m_*	The maximum quantum yield of primary photochemistry at *t* = 0
*ET_0_*/*TR_0_* = 1 − *V_J_*	Probability (at time 0) that a trapped exciton moves an electron into the electron transport chain beyond Q_A_^−^
*ET_0_*/RC = (*M_0_*/*V_J_*) × (1 − *V_J_*)	Electron transport from Q_A_ to Q_B_ flux per RC (reaction center)
*ABS*/RC = [*M_0_* (1/*V_J_*)/(*F_v_*/*F_m_*)]	Absorption flux per RC
*F_v_*/*F_0_*	An indicator of the efficiency in the primary photochemical reaction
Fraction of OEC = [1 − (*V_K_*/*V_J_*)]_treated_/[1 − (*V_K_*/*V_J_*)]_control_	The fraction of OEC (oxygen-evolving complex) in comparison with the control
*DI_0_*/*RC* = *ABS*/*RC* − *TR_0_*/*RC*	Dissipated energy flux per RC at *t* = 0

**Table 2:** EC_50_ of phenol for *M. aeruginosa* and *C. pyrenoidosa* in mono- and co-cultures.

Species	Culture Type	EC_50_ (μg mL^−1^)	Probit Regression Equation *^i^*		
			*a*	*b*	*r* ^2^
*M. aeruginosa*	mono-culture	80.8 ± 0.16	1.16	2.2	0.979
	co-culture	54.7 ± 0.25	1.03	1.58	0.996
*C. pyrenoidosa*	mono-culture	565.9 ± 0.41	0.49	1.36	0.980
	co-culture	NC	NC	NC	NC

*i*: Probit regression equation is in the form of *y* = *ax* + *b*, where *y* = probit (% control), *x* = lg [phenol concentration (in μg mL^−1^)], a is the slope, and b is the y-intercept. NC refers to no calculation because the data were not fit to the dose-response model.

**Table 3:** Logistic equation parameters for two species in mono- and co-cultures.

Species	Culture Type	Phenol Treatment (μg mL^−1^)	*K* (* 10^5^ cells mL^−1^)	*a*	*r*	*R* ^2^	*T_p_* (day)
*M. aeruginosa*	mono-culture	0	408.1	5.73	0.41	0.96	13.9
		2	433.6	5.71	0.41	0.95	13.9
		20	401.2	5.71	0.43	0.98	13.3
		200	128.6	5.78	0.40	0.98	14.5
	co-culture	0	348.8	5.55	0.37	0.98	15.0
		2	268.8	5.17	0.37	0.96	14.0
		20	283.6	5.52	0.38	0.98	14.5
		200	41.4	4.00	0.22	0.95	18.2
*C. pyrenoidosa*	mono-culture	0	281.8	4.25	0.43	0.96	9.8
		2	301.6	4.44	0.45	0.98	9.9
		20	450.6	4.98	0.49	0.98	10.2
		200	433.6	5.36	0.48	0.97	11.2
	co-culture	0	133.4	3.22	0.41	0.95	7.9
		2	139.6	3.25	0.36	0.98	9.0
		20	153.2	3.61	0.37	0.97	9.8
		200	279.1	4.28	0.39	0.92	11.0

Note: *R*^2^ is the determinant coefficient of regression, *K* is the carrying capacity, *a* is a constant, and *r* is the intrinsic growth rate.

**Table 4:** Competitive coefficients of *M. aeruginosa* and *C. pyrenoidosa* in co-cultures.

Phenol Treatment (μg mL^−1^)	*α*	*β*
0	−1.26 ± 0.12	1.75 ± 0.07
2	−0.84 ± 0.08 ^n^	1.58 ± 0.22
20	−0.94 ± 0.18 ^n^	−0.60 ± 0.05
200	−1.12 ± 0.26	−2.19 ± 0.32

Note: *α* is the competitive parameter of *C. pyrenoidosa* against *M. aeruginosa*, while *β* is that of *M. aeruginosa* against *C. pyrenoidosa*; n means there is no significant difference between the treatment and the control.

## Supplementary Materials

The following are available online at https://www.mdpi.com/1660-4601/16/20/3947/s1, Table S1: Changes of the dissolved phenol concentration (μg mL^−1^).
