# Supplementary material for: Effects of Phenolic Pollution on Interspecific Competition between Microcystis aeruginosa and Chlorella pyrenoidosa and their Photosynthetic Responses
Source: Int J Environ Res Public Health. 2019 Oct 17;16(20):3947. doi: 10.3390/ijerph16203947 (PMC6843285; doi:10.3390/ijerph16203947)
Supplement: Supplementary file 1 [file ijerph-16-03947-s001.zip › ijerph-583780-SI.pdf]

Effects of Phenolic Pollution on Interspecific Competition between *Microcystis aeruginosa* and *Chlorella pyrenoidosa* and their Photosynthetic Responses

Supplementary Material

Table S1. Changes of the dissolved phenol concentration (μg mL^{-1}).

	0D	2D	4D	6D	8D	10D	12D	14D	16dD	18D	20D	22D
M. aeruginosa mono-cultures	1.92 ± 0.02	1.88 ± 0.03	1.87 ± 0.02	1.85 ± 0.04	1.82 ± 0.06	1.78 ± 0.07	1.74 ± 0.09	1.71 ± 0.07	1.69±0.06	1.66 ± 0.06	1.64 ± 0.07	1.63 ± 0.10
	19.89 ± 0.03	19.75 ± 0.05	19.63 ± 0.05	19.48 ± 0.08	19.22 ± 0.08	18.83 ± 0.09	18.52 ± 0.10	18.27 ± 0.06	18.03 ± 0.11	17.82 ± 0.12	17.65 ± 0.14	17.53 ± 0.19
	199.90 ± 0.05	199.59 ± 0.06	199.30 ± 0.08	198.93 ± 0.07	198.30 ± 0.16	197.36 ± 0.14	196.60 ± 0.18	195.97 ± 0.14	195.39 ± 0.12	194.89 ± 0.21	194.47 ± 0.30	194.04 ± 0.43
C. pyrenoidosa mono-cultures	1.89 ± 0.03	1.8 3± 0.03	1.80 ± 0.07	1.77 ± 0.06	1.72 ± 0.07	1.65 ± 0.08	1.59 ± 0.09	1.54 ± 0.06	1.50 ± 0.08	1.46 ± 0.08	1.42 ± 0.10	1.39 ± 0.10
	19.87 ± 0.03	19.58 ± 0.09	19.26 ± 0.04	18.19 ± 0.19	18.17 ± 0.12	17.15 ± 0.09	14.58 ± 0.10	13.60 ± 0.15	12.70 ± 0.10	11.92 ± 0.14	11.27 ± 0.15	10.53 ± 0.17
	199.86 ± 0.06	198.70 ± 0.05	197.36 ± 0.06	194.26 ± 0.16	190.53 ± 0.15	188.38 ± 0.13	184.90 ± 0.08	176.73 ± 0.12	173.29 ± 0.18	170.31 ± 0.15	167.82 ± 0.12	165.58 ± 0.20
Co-cultures	1.80 ± 0.03	1.69 ± 0.06	1.65 ± 0.03	1.61 ± 0.09	1.53 ± 0.08	1.42 ± 0.10	1.33 ± 0.07	1.26 ± 0.07	1.19 ± 0.08	1.13 ± 0.07	1.08 ± 0.08	1.06 ± 0.10
	19.75 ± 0.05	19.23 ± 0.06	18.78 ± 0.06	18.85 ± 0.08	17.22 ± 0.13	15.75 ± 0.14	16.34 ± 0.10	15.66 ± 0.14	15.03 ± 0.18	14.49 ± 0.12	14.04 ± 0.06	13.45 ± 0.17
	199.73 ± 0.06	198.21 ± 0.08	196.49 ± 0.07	195.62 ± 0.08	192.72 ± 0.10	184.93 ± 0.15	180.46 ± 0.10	182.00 ± 0.19	179.32 ± 0.23	177.01 ± 0.19	175.07 ± 0.39	173.28 ± 0.32
Blank control (with phenol, without algae)	2.00 ± 0.00	1.99 ± 0.01	1.98 ± 0.01	1.97 ± 0.01	1.96 ± 0.02	1.96 ± 0.02	1.95 ± 0.02	1.93 ± 0.02	1.93 ± 0.02	1.92 ± 0.03	1.91 ± 0.03	1.90 ± 0.02
	20.00 ± 0.00	19.99 ± 0.01	19.98 ± 0.02	19.97 ± 0.02	19.95 ± 0.02	19.95 ± 0.02	19.94 ± 0.02	19.92 ± 0.02	19.90 ± 0.02	19.89 ± 0.02	19.87 ± 0.02	19.85 ± 0.01
	200.00 ± 0.00	199.98 ± 0.02	199.97 ± 0.03	199.96 ± 0.02	199.94 ± 0.03	199.93 ± 0.03	199.93 ± 0.02	199.93 ± 0.03	199.92 ± 0.03	199.91 ± 0.03	199.90 ± 0.03	199.88 ± 0.03
